# Regularities of Encapsulation of Tolfenamic Acid and Some Other Non-Steroidal Anti-Inflammatory Drugs in Metal-Organic Framework Based on γ-Cyclodextrin

**DOI:** 10.3390/pharmaceutics15010071

**Published:** 2022-12-26

**Authors:** Ekaterina Delyagina, Anna Garibyan, Mikhail Agafonov, Irina Terekhova

**Affiliations:** 1 Institute of Mathematics, Information Technology and Natural Sciences, Ivanovo State University, 153025 Ivanovo, Russia; 2 G.A. Krestov Institute of Solution Chemistry of RAS, 153045 Ivanovo, Russia

**Keywords:** metal-organic frameworks, γ-cyclodextrin, tolfenamic acid, non-steroidal anti-inflammatory drugs, encapsulation, release, permeability

## Abstract

Metal-organic frameworks based on cyclodextrins (CDs) have been proposed as promising drug delivery systems due to their large surface area, variable pore size, and biocompatibility. In the current work, we investigated an incorporation of tolfenamic acid (TA), a representative of non-steroidal anti-inflammatory drugs (NSAIDs), in a metal-organic framework based on γ-cyclodextrin and potassium cations (γCD-MOF). Composites γCD-MOF/TA obtained by absorption and co-crystallization methods were characterized using powder X-ray diffraction, low temperature nitrogen adsorption/desorption, scanning electron microscopy, and FTIR spectroscopy. It was demonstrated that TA loaded in γCD-MOF has an improved dissolution profile. However, the inclusion of TA in γ-CD reduces the membrane permeability of the drug. A comparative analysis of the encapsulation of different NSAIDs in γCD-MOF was performed. The impact of NSAID structure on the loading capacity was considered for the first time. It was revealed that the presence of heterocycles in the structure and drug lipophilicity influence the loading efficiency of NSAIDs in γCD-MOF.

## 1. Introduction

As is well known, a large number of drugs are hydrophobic in nature and, therefore, poorly soluble in aqueous media. To increase the aqueous solubility and bioavailability of these poorly soluble drugs, numerous solubilization techniques such as solid dispersions [1,2], micronization [3], inclusion complex formation [4], micellar solubilization [5], hydrotropy [6], and co-crystallization [7] have been developed and used. In our work, particular attention was focused on cyclodextrins (CDs), which are naturally occurring cyclic oligosaccharides obtained from starch by enzymatic conversion. Cyclodextrins have been widely used to improve the solubility and stability of numerous drugs and biologically active compounds [8]. It is known that the CD molecules have a hydrophilic outer surface and a hydrophobic inner cavity, which facilitates the binding and solubilization of lipophilic guest molecules in an aqueous media [9,10].

Recently, in the chemistry of CDs a new direction has appeared, associated with the design of more complicated supramolecular systems such as metal-organic frameworks (MOFs) [11,12]. Metal-organic frameworks are hybrid crystalline porous materials consisting of organic molecules coordinated by metal ions. Cyclodextrins can be successfully employed as linkers in these supramolecular structures [11,12,13,14,15]. Metal-organic frameworks based on cyclodextrins (CD-MOF) are biocompatible crystalline structures with a highly developed specific surface area. Due to the presence of pores of different diameter, CD-MOFs are able to accommodate various substances, acting not only as carriers, but also as an effective solubilizers [11,12,16,17,18,19,20,21,22]. For example, it has been demonstrated that γ-CD-based MOFs (γCD-MOF) can serve as superior carriers for curcumin [17,18], ascorbic acid derivatives [19], hexanal [20], methotrexate [21], and leflunomide [22].

**Figure 1:**
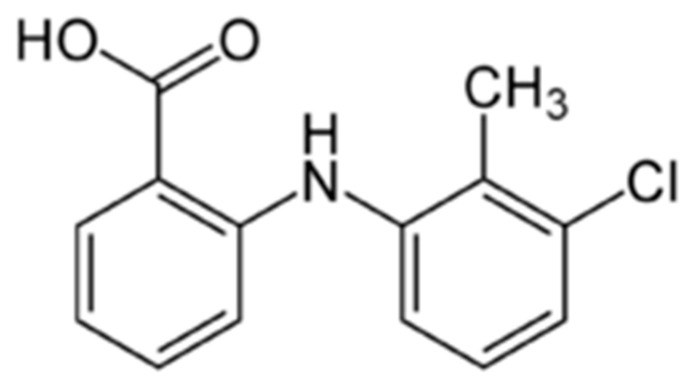
Structural formula of tolfenamic acid.

To the best of our knowledge, a number of non-steroidal anti-inflammatory drugs (NSAIDs) have been loaded in CD-MOFs [23,24,25,26,27,28]. Non-steroidal anti-inflammatory drugs are one of the most popular and commonly applied classes of medication widely used to relieve pain and reduce inflammation. The low aqueous solubility and unwanted side effects of NSAIDs encourage the design of the drug delivery systems [29]. As is known from the literature, CD-MOFs can be a suitable platform for the loading of ibuprofen [23,24], diclofenac [25], fenbufen [26], ketoprofen [27], and flurbiprofen [28]. In our recent work, the number of encapsulated NSAIDs was increased—niflumic acid was loaded in γCD-MOF by absorption and co-crystallization methods [30]. In the current research, tolfenamic acid (TA, Figure 1) belonging to the fenamates group, a subgroup of NSAIDs, was immobilized in γCD-MOF. Tolfenamic acid is primarily used to reduce the pain associated with acute migraine attack as well as the swelling in muscles and joints occurring in osteoarthritis and rheumatoid arthritis [31,32]. Tolfenamic acid is insoluble in aqueous solutions [33,34], which complicates the preparation of the dosage forms. In particular, the solubility of TA is extremely low (13.6 nM) in acidic media [34]. Cafaggi et al. [33] reported that TA is practically insoluble in water (~2 × 10^−6^ M at 25 °C).

Therefore, some attempts to increase TA solubility have been made [33,34,35,36,37,38,39]. It has been demonstrated that TA solubility can be improved by complexation with CDs [35,36,37,38] and poloxamers [33] as well as by the formation of sodium salts and multicomponent crystals [34]. In particular, the solubility of TA in the presence of hydroxypropyl-β-CD and methyl-β-CD was increased by 30 and 2 times, respectively [34]. Poloxamer P407 was a more effective solubilizer; at a concentration of 12 wt.% it induced a 2000-fold rise of TA solubility in the aqueous solution at 25 °C [33]. Tolfenamic acid loaded in high molecular weight polyethylene displayed improved properties [39].

In this work, γCD-MOF was proposed as a carrier for TA. Tolfenamic acid was loaded in γCD-MOF by absorption and co-crystallization methods. The obtained composites were characterized using powder X-ray diffraction (PXRD), low temperature nitrogen adsorption/desorption, scanning electron microscopy (SEM), and FTIR spectroscopy methods. The improvement of the pharmacologically important properties of TA immobilized in γCD-MOF was revealed.

The NSAIDs under study have different structures and properties. Therefore, it was interesting to reveal the main regularities demonstrating the influence of the NSAID structure on the loading efficiency in γCD-MOF. This relationship was established for the first time on the basis of the comparative analysis of our own experiments, and is available in the literature [24,26,27,28,30] data. The revealed relationship could be a predictive tool in the design of drug delivery systems for NSAIDs. According to [27], γCD-MOFs were found to be not cytotoxic (cell viability mean > 100%), indicating good biocompatibility.

## 2. Materials and Methods

TA, γ-CD and KOH were purchased from Sigma-Aldrich (Russia, Moscow) and used as received. All reagents used for preparation of the buffers (HCl, KCl, Na_2_HPO_4_, NaOH) and ethanol were of analytical grade. The pH of the solutions was controlled by means of Five Easy pH-meter (Mettler Toledo, Columbus, OH, USA).

γCD-MOF was synthesized from the aqueous solution of γ-CD and KOH taken at 1:8 molar ratio by the vapor diffusion method according to the protocol reported by Smaldone et al. [11]. The obtained crystalline samples were separated from the mother liquor, washed with methanol, and activated by drying under vacuum at 80 °C for 15 h to remove the residual solvent.

The TA was loaded in γCD-MOF by absorption and co-crystallization methods. The first method was as follows. The activated γCD-MOF (0.15 g) was soaked in ethanol solution (10 mL) of TA (0.005 M). The mixture was shaken on Eppendorf ThermoMixer C at 25 °C during 48 h. The co-crystallization is similar to the protocol of γCD-MOF synthesis from the solution of γ-CD and KOH (taken at 1:8 molar ratio) with TA (0.05 M). The γCD-MOF samples loaded by TA were collected, washed with ethanol, and dried under vacuum at 80 °C for 15 h. The drug-loading capacity was calculated as

(1) $$w=\frac{g\left(TA\right)}{g\left(\gamma CD-MOF/TA\right)}\cdot 100\%$$

where *g*(γCD-MOF/TA) is amount of the composite; *g*(TA) is the amount of TA in the composite that was determined spectrophotometrically (spectrophotometer Shimadzu UV-1800, Kyoto, Japan, Figure S1).

Adsorption of TA on γCD-MOF was studied in detail. To this end, γCD-MOF was added to ethanolic solution of TA. The samples were shaken on Eppendorf ThermoMixer C at 25 °C. The adsorption equilibrium study was performed in the TA concentration range from 200 to 1200 mg/L. To investigate the adsorption kinetics, the initial concentration of TA was constant and supernatant was collected at specific time intervals. The samples were centrifuged for 20 min at 12,000 rpm (Thermo Scientific MicroCL 21R) and supernatant was analyzed spectrophotometrically (Shimadzu UV-1800). Equations (2) and (3) were used to calculate the amount of adsorbed TA:

(2) $$Q_{e}=\frac{\left(C_{0}-C_{e}\right)\cdot V}{g}\;$$

(3) $$Q_{t}=\frac{\left(C_{0}-C_{t}\right)\cdot V}{g}$$

where *C*_0_ is initial concentration of TA; *C_e_* is TA concentration at equilibrium; *C_t_* is TA concentration at *t* min after adsorption; *V* is the initial volume of the TA solution; *g* is the mass of γCD-MOF.

Afterward, the obtained composites γCD-MOF/TA were characterized by different physicochemical methods. Powder X-ray diffraction analysis was performed using a Bruker D8 Advance diffractometer (CuK α = 1.54 Å). The N_2_ adsorption/desorption isotherms were obtained by means of a porosity analyzer Nova Series 1200e (Quantachrome, Boynton Beach, FL, USA). The FTIR measurements were carried out on a VERTEX 80v FTIR spectrometer (Bruker, Germany). Samples under study were ground with IR grade KBr, and the spectra were scanned over a frequency range of 350–4000 cm^−1^. Scanning electron microscopy analysis was performed to analyze the morphology of the samples using a scanning electron microscope Quattro S (Thermo Fisher Scientific, Czech Republic).

Release of TA from the composites in aqueous buffers was investigated on the dissolution tester (LanIndia DS 8000) using a basket method. Temperature of the dissolution system (37 ± 0.5 °C) and stirring rate (50 rpm) were kept constant. Buffers simulating the gastric fluid (pH 1.6, HCl) and intestinal fluid (phosphate buffer pH 6.8) were employed as the dissolution media. At given time intervals, 0.2 mL of solution was withdrawn for analysis by UV–vis absorption (Shimadzu UV-1800) and replaced by the same volume of buffer.

In vitro permeability study was performed using a vertical Franz diffusion cell (PermeGear, Hellertown, PA, USA). The acceptor compartment was filled with phosphate buffer (pH 7.4) simulating the bloodstream; the donor compartment was filled with TA solution. Regenerated cellulose membrane (molecular weight cut-off is 12 kDa) was used as a model membrane. The experiments were conducted at constant temperature (37 °C). Aliquots of 0.5 mL were withdrawn from the acceptor compartment and replaced with the equivalent volume of the buffer. Concentration of TA was determined spectrophotometrically (Shimadzu UV-1800). The apparent permeability coefficient (*P_app_*) was calculated as follows:

(4) $$P_{app}=\frac{Q}{A\cdot C_{0}}$$

where *Q* is the steady-state appearance rate of TA (mol/s); *A* is the surface area of the membrane (cm^2^); *C*_0_ is initial concentration of TA in the donor compartment (mol/L).

Additionally, the solubilizing effect of γ-CD towards TA in buffers (pH = 1.6 and pH = 6.8) was studied by the shake-flask method. To this end, an excess amount of TA was added to vials containing solutions of γ-CD of variable concentration. The resulting suspensions were shaken on Eppendorf ThermoMixer C at 25 °C for 72 h to ensure that the solubility equilibrium was reached. Then, the undissolved TA was removed by centrifugation at 25 °C (Thermo Scientific MicroCL 21R), and the clear saturated solution was used to determine the solubility using spectrophotometer Shimadzu UV-1800. Binding constants (*K*) of TA with γ-CD were calculated from the slope of the obtained phase solubility diagrams using Higuchi and Connors approach [40]:

(5) $$K=\frac{slope}{S_{0}\cdot \left(1-slope\right)}$$

where *S*_0_ is solubility of TA in buffer without γ-CD.

## 3. Results and Discussion

### 3.1. Characterization of Composites γCD-MOF/TA

**Figure 2:**
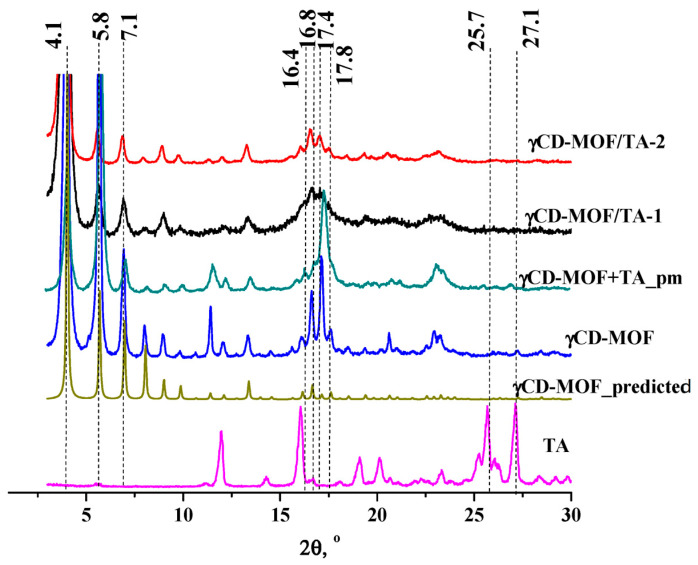
The PXRD patterns of the samples.

γCD-MOF was synthesized from γ-CD and KOH by the vapor diffusion method proposed in [11]. After the synthesis, the structure of γCD-MOF was confirmed using PXRD and N_2_ adsorption/desorption measurements. The PXRD results indicated that all the characteristic diffraction lines originating from the well-described crystal structure of the γCD-MOF [11] were observed in the diffraction pattern at 4.1, 5.8, 7.1, 16.4, 16.8, 17.4, and 17.8° (Figure 2). This result evidences the phase purity of the synthesized γCD-MOF. According to Smaldone et al. [11], six γ-CDs are coordinated by alkali metal cations on the primary face forming (γ-CD)_6_ cubes, which are linked together in three dimensions by the coordination of K^+^ cations on the secondary faces of the γ-CD tori. As a result, the porous structure, which is schematically shown in Figure S2, is formed.

**Figure 3:**
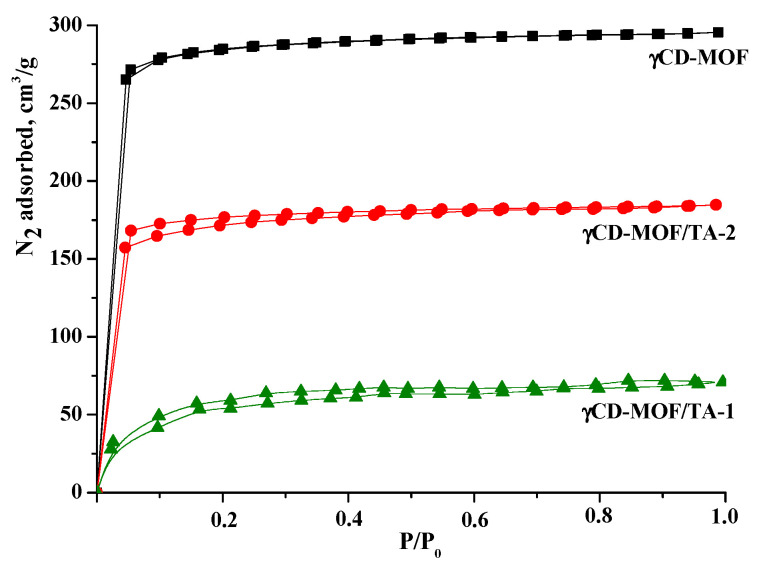
Nitrogen absorption/desorption isotherms for γCD-MOF, γCD-MOF/TA-1, and γCD-MOF/TA-2.

The N_2_ absorption/desorption isotherms are shown in Figure 3. As one can see, γCD-MOF demonstrated type I isotherm patterns corresponding to microporous materials. According to calculations using the Brunauer–Emmett–Teller (BET) method, the surface area of γCD-MOF was 833 m^2^/g, which was in a good agreement with the literature data (820 m^2^/g [41] and 816 m^2^/g [27]).

Tolfenamic acid was loaded in γCD-MOF by the absorption and co-crystallization methods. The composites obtained by these two methods were designated as γCD-MOF/TA-1 and γCD-MOF/TA-2, respectively. The amount of TA in γCD-MOF/TA-1 and γCD-MOF/TA-2 was measured spectrophotometrically as 7 wt.% and 5.4 wt.%, respectively. The theoretical loading of TA in γCD-MOF was 8.7 wt.% and 18.7 wt.% for γCD-MOF/TA-1 and γCD-MOF/TA-2, respectively.

The PXRD analysis of γCD-MOF/TA-1 and γCD-MOF/TA-2 clearly showed the presence of all the characteristic diffraction lines of γCD-MOF in the diffraction patterns of both composites (Figure 2). This result suggests that the crystalline nature of γCD-MOF was kept after TA loading. γCD-MOF contributes more to the PXRD trace than TA. It should be noted that the characteristic diffraction lines of TA do not appear on the diffractograms of the composites. It seems that TA loaded in γCD-MOF does not form the crystal phase in the framework. However, the absence of the characteristic diffraction lines of TA on the diffractograms of the composites could be due to the low TA content (5–7 wt.%). To verify this assumption, PXRD measurements were performed for the physical mixture of γCD-MOF with TA (7 wt.%). It is evident from Figure 2 that the characteristic diffraction lines of TA at 25.7° and 27.1° are observed in the diffraction pattern of the physical mixture, whereas they are not visible in the diffractograms of the composites. There are some other differences in the PXRD patterns of the physical mixture and composites. Namely, the intensity of the main diffraction lines of γCD-MOF in the diffraction patterns of the intact γCD-MOF and physical mixture (γCD-MOF + TA) is very close, while the intensity of the diffraction lines at 11.4° and 17.1° is considerably decreased in the case of the composites.

The comparison of the diffraction patterns of the composites shows that γCD-MOF/TA-1 has a slightly lower crystallinity than γCD-MOF/TA-2. We suppose that the crystallinity of γCD-MOF/TA-1 was disturbed in the ethanolic solution of TA. As has been documented in the literature [23,24], the CD-MOF crystallinity was impaired during the loading in ethanol. However, in spite of this, ethanol is widely used as the solvent during absorption since the loading increases significantly [23].

Scanning electron microscopy analysis was performed to investigate the surface morphology of the empty and loaded γCD-MOF. Figure S3 shows the SEM images of γCD-MOF, γCD-MOF/TA-1, and γCD-MOF/TA-2. The empty γCD-MOF consists of cubic crystals. The cubic morphology of the crystals is well defined for the composite γCD-MOF/TA-2. However, some crystals of γCD-MOF/TA-1 were damaged due to the mechanical agitation during the loading.

The nitrogen absorption/desorption isotherms obtained before and after TA loading are illustrated in Figure 3. As one can see, the inclusion of TA in γCD-MOF induces the decrease in surface area in the following order: γCD-MOF (833 m^2^/g) > γCD-MOF/TA-2 (512 m^2^/g) > γCD-MOF/TA-1 (189 m^2^/g). The reduction in surface area could be explained by the presence of the encapsulated TA. The revealed decrease in porosity is in agreement with the TA content in the composites. According to Smaldone at al. [11], pores of different diameters are available in γCD-MOF. They are large spherical pores (1.7 nm), pore windows (0.78 nm), and infinite pores (0.42 nm). The approximate geometric dimensions of TA are given in Figure S4. It seems that spherical pores are more receptive to TA incorporation.

**Figure 4:**
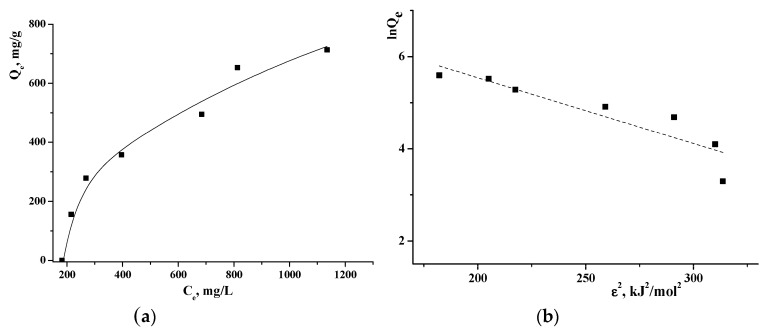
Adsorption isotherm of TA on γCD-MOF (**a**) and Dubinin–Radushkevich plot (**b**) at 25 °C.

To study the sorption of TA on γCD-MOF, the adsorption isotherm was obtained by varying the initial TA concentration. As seen from the adsorption isotherm (Figure 4a), the amount of the adsorbed TA increased with the rise of TA concentration while the active sites were saturated. To study the adsorption mechanism, Langmuir, Freundlich, Temkin, and Dubinin–Radushkevich adsorption models were used. The value of the correlation coefficient *R*^2^ (Table S1) was applied to measure the degree of compliance with the adsorption model. It was found that the Temkin model had the highest fitting degree. The Temkin isotherm model postulates that the adsorption process is characterized by a uniform distribution of binding energies at the adsorbent surface [42]. This model indicates the exothermic nature of the adsorption reaction as a slope > 0, which is an indicator of heat release during the process [43]. The Temkin isotherm contains a factor that explicitly takes into account the adsorbent–adsorbate interactions.

The Dubinin–Radushkevich isotherm is generally applied to express the adsorption mechanism and to differentiate between the physical and chemical adsorption. The linearized form of the Dubinin–Radushkevich equation is given as:

(6) $$lnQ_{e}=lnQ_{max}-K_{DR}\varepsilon^{\;2}$$

where *K_DR_* is the Dubinin–Radushkevich isotherm constant (mol^2^/J^2^); *ε* is the adsorption potential (J/mol), which can be written as:

(7) $$\varepsilon =RTln\left(1+\frac{1}{c_{e}}\right)$$

where *T* is the temperature (K); *R* is the gas constant (J/(mol K)); *c_e_* is the equilibrium concentration of TA (M). The values of *Q_max_* and *K_DR_* are evaluated from the intercept and the slope of the plot of ln*Q_e_* versus *ε*^2^ (Figure 4b).

The mean adsorption energy *E* can be obtained by the following equation:

(8) $$E=\frac{1}{\sqrt{2K_{DR}}}$$

It is believed [44] that the magnitude of *E* indicates the type of adsorption: if *E* < 8 kJ/mol, the adsorption proceeds physically; if *E* > 8 kJ/mol, the adsorption process occurs chemically. The obtained *E* = 5.9 kJ/mol points out that TA is immobilized in γCD-MOF by the physical adsorption.

**Figure 5:**
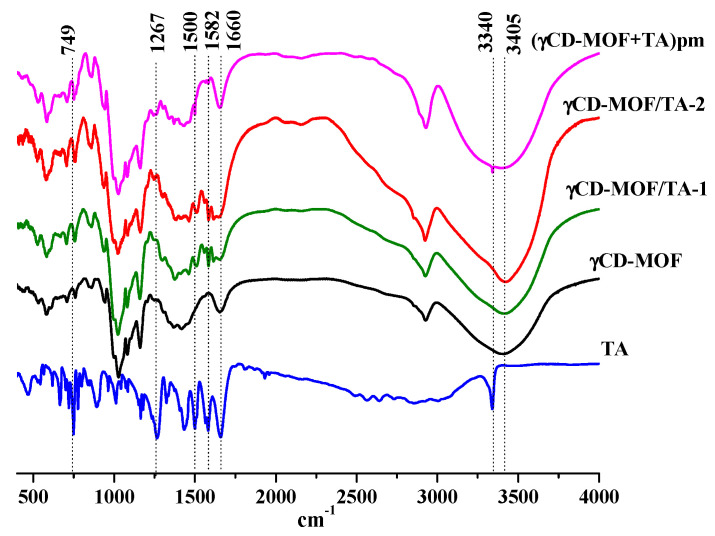
**The** FTIR spectra of the samples.

The physical adsorption of TA was confirmed by FTIR spectroscopy. The FTIR spectra of TA, γCD-MOF, γCD-MOF/TA-1, γCD-MOF/TA-2, and the physical mixture (γCD-MOF + TA) are given in Figure 5. The FTIR spectrum of TA exhibited the characteristic bands at 3340 cm^−1^ for the stretching vibration of the amino group and at about 1500 cm^−1^ for its bending vibration [45]. The carbonyl and benzene ring stretching vibrations are exhibited at 1660 cm^−1^ and 1582 cm^−1^, respectively. The bands at 1267 cm^−1^ and 749 cm^−1^ are attributed to C-H and C-N deformation, respectively [45]. All these bands are clearly observed in the FTIR spectrum of the physical mixture of γCD-MOF with TA. However, in the FTIR spectra of the composites (γCD-MOF/TA-1 and γCD-MOF/TA-2), the band at 3340 cm^−1^ from the stretching vibration of the amino group disappeared; the absorption band at 1660 cm^−1^, due to the carbonyl stretching vibration, was less pronounced; the band corresponding to the bending vibration of the amino group was shifted from 1500 cm^−1^ to 1512 cm^−1^; the band at 749 cm^−1^, owing to C-N deformation, disappeared or was overlapped with the band from γCD-MOF. Moreover, the band at 1582 cm^−1^ became more visible and the band at 1267 cm^−1^ was shifted to 1288 cm^−1^ in the spectra of the composites. The observed changes in the FTIR spectra indicate interactions of TA with γCD-MOF in the composites. We suppose the possibility of intermolecular hydrogen bonding between γ-CD hydroxyls and polar groups (-COOH and -NH) of TA. This assumption is supported by the changes of the characteristic band of γCD-MOF at 3405 cm^−1^ corresponding to the -OH stretching of the cyclodextrin moiety. This band was narrower in the spectra of the composites compared with the spectrum of the physical mixture. The revealed difference is due to the participation of the -OH groups of γCD-MOF in the H-bonding with TA in the composites.

### 3.2. Pharmacologically Important Properties of TA Loaded in γCD-MOF

**Figure 6:**
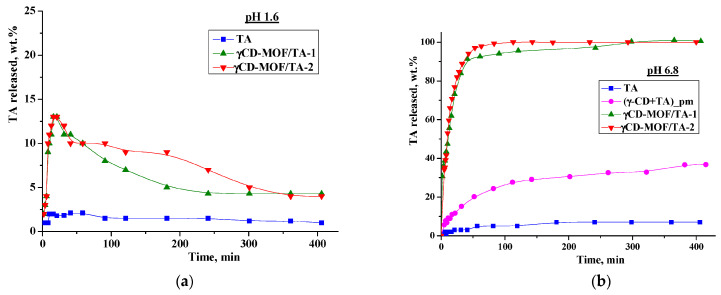
Release profiles of TA at 37 °C ((**a**)—at pH = 1.6; (**b**)—at pH = 6.8).

We performed in vitro drug release experiments in the model media simulating the gastric fluid (pH = 1.6) and the intestinal fluid (pH = 6.8). The release profiles are shown in Figure 6. As one can see from Figure 6a, TA displays very slow dissolution in buffer with pH = 1.6—less than 2% was dissolved during 7 h. The immobilization of TA in γCD-MOF accelerates the dissolution process—a burst release of 13 wt.% during the first 20 min was detected for both composites. After that, we observed the smoothly decreasing TA concentration in the dissolution medium due to the precipitation (Figure 6a). Nevertheless, the concentration of TA released from the frameworks was higher than the concentration of raw TA. In the literature [46,47], this phenomenon is called the “spring and parachute effect”. We suppose that the dissolution of the composites in aqueous medium is accompanied by the collapse of the framework. In this case, γ-CD, which is more soluble (0.18 M) compared with TA, is drawn out into the aqueous medium, and in such a medium TA becomes supersaturated. This supersaturated state is maintained for a sufficient period of time.

**Figure 7:**
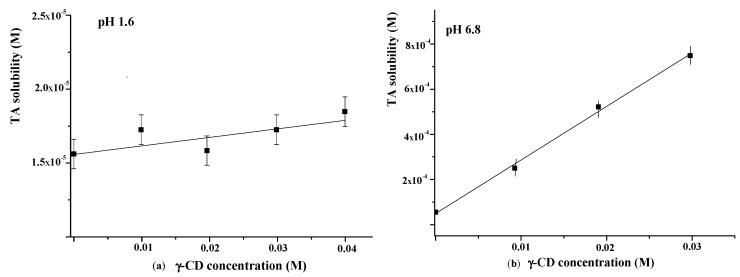
Phase solubility diagrams of TA in the presence of γ-CD at 25 °C ((**a**)—at pH = 1.6; (**b**)—at pH = 6.8).

The influence of γ-CD on TA solubility in the buffer with pH = 1.6 was additionally investigated using the Higuchi and Connors approach [40]. The obtained phase solubility diagram is depicted in Figure 7a. As one can see from Figure 7a, the TA solubility in buffer (pH = 1.6) is very low (1.6 × 10^−6^ M). A slight increase in TA solubility with the rise of γ-CD concentration was detected. The binding constant derived from the slope of the solubility diagram was very low (*K* = 4 M^−1^), indicating the weak binding affinity of γ-CD to TA in the acidic medium.

As is evident from Figure 6, the release behavior of TA in the buffer with pH = 1.6 and the buffer with pH = 6.8 is different. Namely, the released amount of pure TA at pH = 6.8 is considerably higher and represents approximately 7 wt.% after 7 h (Figure 6b). The revealed difference is due to the TA ionization in the phosphate buffer (p*K_a_* = 3.7 [48]). Owing to ionization, the TA solubility in the buffer with pH 6.8 is also higher (5.5 × 10^−5^ M) compared with the buffer with pH = 1.6 (Figure 7). Thus, the improved solubility and dissolution rate of TA at pH = 6.8 are due to the stronger affinity of the ionized TA to the aqueous environment compared with the TA molecular form existing at pH = 1.6. In the phosphate buffer (pH = 6.8), the TA loaded in γCD-MOF has a faster release (100 wt.% at 1.5 h) in comparison with the pure TA (5 wt.% at 1.5 h). We suppose that TA is included in γCD-MOF in a molecular state, the release of which is accelerated [27]. This factor is mainly responsible for the observed fast release of TA from the composite γCD-MOF/TA.

To confirm this assumption, the release of TA from the physical mixture with γ-CD was studied additionally. The physical mixture was prepared from the intact TA and γ-CD taken in a ratio of 6:94 (wt.%), which is close to the content of the composites. It is evident from Figure 6b that the release rate in the buffer with pH = 6.8 is increased for the physical mixture compared with the pure TA, but at the same time, it is considerably lower compared with the composite γCD-MOF/TA. In solution, TA with γ-CD forms stable water-soluble inclusion complexes (*K* = 473 M^−1^), as was supported by the solubility data (Figure 7b). Thus, the inclusion complex formation of TA with γ-CD occurring in the solution could be responsible for the observed increased release rate.

To further analyze the release kinetics of the composites in the buffer with pH 6.8, the zero-order, first-order, Hixson–Crowell, and Korsmeyer–Peppas kinetic models were applied (Table S2). The best-fit determines the model that describes the drug release profile (Figure 6) by the highest correlation coefficient (R^2^). It was found that the Korsmeyer–Peppas model showed the best fitting ability (Table S2). This kinetic model is based on the idea that the release of the substance included in matrices proceeds not only by its diffusion from the carrier into the bulk of the solution, but also due to the destruction of the carrier itself [49]. The *n* value derived from the Korsmeyer–Peppas model represents the drug release mechanism. For the Fickian diffusion mechanism, *n* < 0.43, while 0.43 < *n* < 0.85 corresponds to non-Fickian drug release behavior [50]. For γCD-MOF/TA-1 and γCD-MOF/TA-2, the values of *n* were 0.46 and 0.43, respectively, (Table S2) indicating the combination of TA diffusion and the γCD-MOF erosion mechanism.

**Figure 8:**
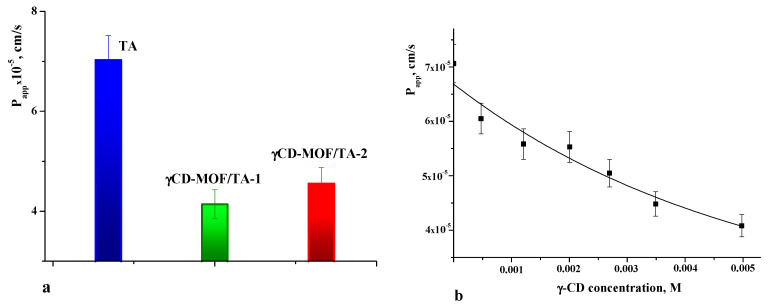
Apparent permeability coefficients of TA (**a**) and dependence of *P_app_* on γ-CD concentration (**b**) in phosphate buffer pH = 7.4 at 37 °C.

As is well known, the therapeutic efficiency of drugs also depends on their ability to cross the cell membrane. Therefore, membrane permeability is the next key property to consider during the drug design process [51]. The permeability coefficients were determined for pure TA and TA loaded in γCD-MOF. The values of *P_app_* are given in Figure 8a. The comparative analysis of *P_app_* shows that the permeability of the TA released from the framework is lower compared with the intact TA. The observed decrease in *P_app_* for the TA released from the composites is attributed to the inclusion complex formation with γ-CD. In solution, the framework collapses and TA interacts with γ-CD. Thus, pure TA and γ-CD/TA complexes coexist in solution. However, the permeation of the complexes across the membrane is limited by their slow diffusion caused by the larger size and higher viscosity of the solution. As a result, *P_app_* decreases in the case of the composites.

To further elucidate the effect of γ-CD complexation on the permeability of TA, the concentration dependence of *P_app_* was obtained (Figure 8b). As seen from Figure 8b, the permeability of the TA across the cellulose membrane was decreased with the increasing concentration of γ-CD. This correlates with the complex formation, which gives the rise of the fraction of the inclusion complexes in the solution. Thus, this fact should be taken into account when considering the impact of γCD-MOF on the pharmacologically important properties of TA.

### 3.3. Comparative Analysis of the Absorption of NSAIDs in γCD-MOF

**Figure 9:**
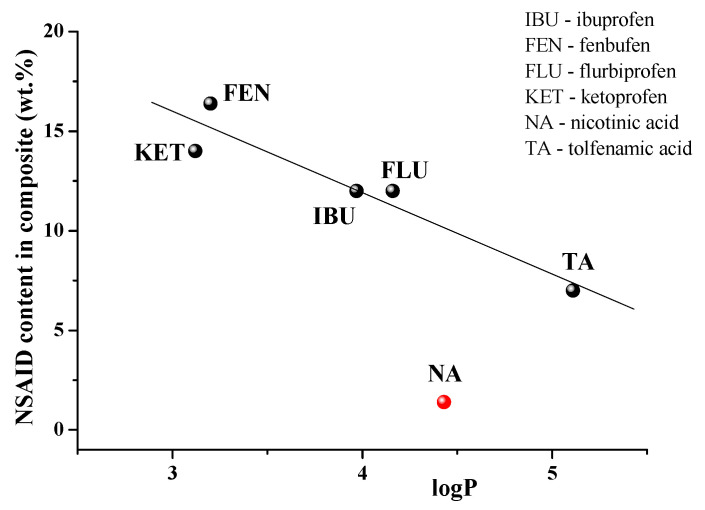
NSAIDs loading efficiency versus log*P*.

To date, there are a number of publications devoted to the immobilization of NSAIDs in γCD-MOF [23,24,25,26,27,28]. The loaded NSAIDs (TA, niflumic acid [30], ibuprofen [23,24], ketoprofen [27], fenbufen [26], flurbiprofen [28]) have different structures and physicochemical properties (Table S3). Therefore, it was interesting to analyze the influence of these factors on the efficiency of the drug loading in γCD-MOF. To reveal the main regularities of the entrapment of NSAIDs in γCD-MOF, we considered the composites obtained by the absorption in ethanol (γCD-MOF/NSAID-1). After analyzing the impact of the lipophilicity, the polar surface area of the molecules, and the ability to ionize we found the dependence of drug loading percentages only on log*P*. As follows from the dependence depicted in Figure 9, the inclusion of ketoprofen and fenbufen as more lipophilic drugs is favorable compared with the more hydrophilic TA and NA.

As one can see from Figure 9, niflumic acid does not belong to the revealed dependence. A characteristic feature of niflumic acid is the availability of the pyridine ring in the structure. Probably, it restricts the immobilization of the drug into the pores of γCD-MOF. This result is in accordance with the available literature data. Namely, Liu et al. [28], considering the insertion of drugs of different classes in CD-MOFs, demonstrated that most of the drug molecules containing nitrogen-containing heterocyclic rings showed relatively low adsorption (<5%). Thus, the proposed regularity is true for the encapsulation of NSAIDs in γCD-MOF.

## 4. Conclusions

In summary, γCD-MOF was considered a promising delivery system for NSAIDs. It was demonstrated that TA could be successfully immobilized in γCD-MOF by absorption and co-crystallization methods. The adsorption energy obtained from the Dubinin–Radushkevich isotherm suggests that the uptake of TA in γCD-MOF was by physisorption. However, the possibility of hydrogen bonding between γ-CD hydroxyls and polar groups (-COOH and -NH) of TA is not excluded. Loading in γCD-MOF results in the significant improvement of the TA release in the biorelevant media. At the same time, the membrane permeability of TA was decreased due to the complex formation with γ-CD.

γCD-MOF was considered a supramolecular platform for the delivery of NSAIDs of different structures and properties. The main regularities of the encapsulation of NSAIDs in γCD-MOF were proposed for the first time. The dependence of the loading efficiency on the lipophilicity of NSAIDs was revealed. The inclusion of nitrogen-containing heterocyclic structures in porous γCD-MOF was shown to be not preferable.

## Data Availability

Not applicable.

## Supplementary Materials

The following supporting information can be downloaded at: https://www.mdpi.com/article/10.3390/pharmaceutics15010071/s1, Table S1: Models for adsorption isotherms; Table S2: Kinetic models of drug release; Table S3: Summary of NSAIDs loaded in γCD-MOF by absorption in ethanol and lipophilicity coefficients of NSAIDs; Figure S1: SEM images of γCD-MOF (a), γCD-MOF/TA-1 (b), and γCD-MOF/TA-2 (c); Figure S2: Schematic representation of γCD-MOF (a), γCD-MOF/TA (b) and inclusion complex γ-CD/TA (c); Figure S3: SEM images of γCD-MOF (a), γCD-MOF/TA-1 (b) and γCD-MOF/TA-2 (c); Figure S4: Geometric dimensions of TA.
