# Supplementary material for: Regularities of Encapsulation of Tolfenamic Acid and Some Other Non-Steroidal Anti-Inflammatory Drugs in Metal-Organic Framework Based on γ-Cyclodextrin
Source: Pharmaceutics. 2022 Dec 26;15(1):71. doi: 10.3390/pharmaceutics15010071 (PMC9867401; doi:10.3390/pharmaceutics15010071)
Supplement: Supplementary file 1 [file pharmaceutics-15-00071-s001.zip › pharmaceutics-2066450-supplementary.pdf]

SUPPORTING INFORMATION

Regularities of encapsulation of tolfenamic acid and some other non-steroidal anti-inflammatory drugs in metal organic frameworks based on γ-cyclodextrin

Ekaterina Delyagina [a,*], Anna Garibyan [b], Mikhail Agafonov [b], Irina Terekhova [b]

[a]*Institute of Mathematics, Information Technology and Natural Sciences, Ivanovo State University*

[b]*G.A. Krestov Institute of Solution Chemistry of RAS*

Table S1. Models for adsorption isotherms

Model	Equation	R^2
Langmuir	$$\frac{1}{Q_e} = \frac{1}{Q_m \cdot b} + \frac{1}{C_e}$$ where Q_e (mg·g^{-1}) - is the amount of TA that can be absorbed by a unit mass of the adsorbent at equilibrium; C_e (mg·L^{-1}) – is the concentration of TA in solution at equilibrium; Q_m – is the maximum adsorption capacity (mg·L^{-1}); b (L·mg^{-1}) – is the Langmuir constant, which is related to the affinity between the adsorbent and adsorbate.	0.020
Freindlich	$$logQ_e = logk_F + \frac{1}{n} logC_e$$ where k_F ((mg/g)/(mg/L)$^{1/n}$) – is the constant related to the sorption capacity of the adsorbent, indicative of the affinity between the species; *n* is the constant related to the sorption intensity.	0.454
Temkin	$$Q_e = \frac{RT}{b_T} lnK_T + \frac{RT}{b_T} lnC_e$$ where b_T – is the Temkin constant related to the heat of adsorption (J·mol^{-1}); K_T – is the Temkin isotherm equilibrium binding constant (L·g^{-1}); R – is the gas constant (8.314 J·mol^{-1}·K^{-1}); T – is the temperature (K).	0.961

Table S2. Kinetic models of drug release

Model	Equation	R^2	
		γCD-MOF/TA-1	γCD-MOF/TA-2
Zero order	$C = K_0t$<br>K_0 is zero order rate constant, t is the time	0.462	0.429
First order	$\log C_t = \log C_0 - k_1t/2.303$<br>$C_t$ is amount of drug released in t time;<br>C_0 is the initial amount of drug	0.412	0.355
Hixson-Crowell	$Q_0^{1/3} – Q_t^{1/3} = K_{HC}t$<br>$Q_0$ is the initial amount of drug; Q_t is the amount of drug remained after t time; K_{HC} is Hixson-Crowell dissolution rate constant	0.462	0.380
Korsemeyer-Peppas	$\log(M_t/M_\infty) = \log k + n\log t$<br>k is dissolution rate constant; n is release exponent;<br>M_t/M_∞ is the drug release fraction in the media	0.979<br>(n=0.46)	0.925<br>(n=0.43)

Table S3. Summary of NSAIDs loaded in γCD-MOF by impregnation in ethanol and lipophilicity coefficients of NSAIDs

**Name**	**Structural formula**	**Loaded amount (wt.%)**	**log*P*[*]**
Tolfenamic acid (TA)	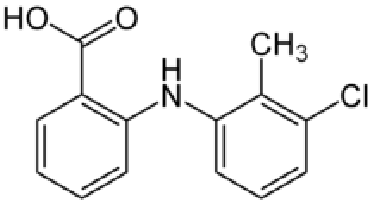	7	5.17
Niflumic acid (NA)	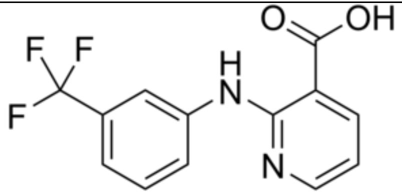	1.4 [30]	4.43
Ibuprofen (IBU)	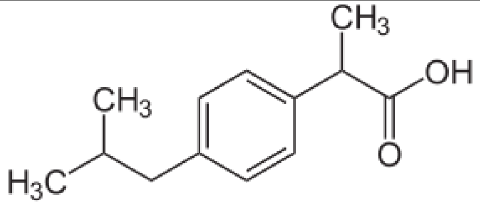	12 [24]	3.97
Fenbufen (FEN)	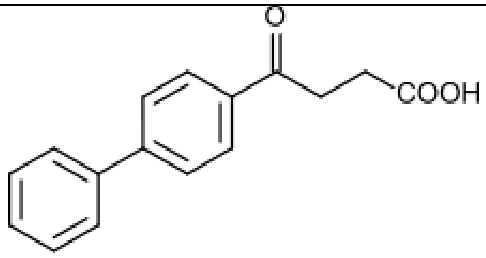	16.4 [26]	3.2
Ketoprofen (KET)	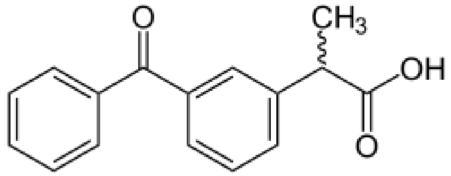	14 [27]	3.12
Flurbiprofen (FLU)	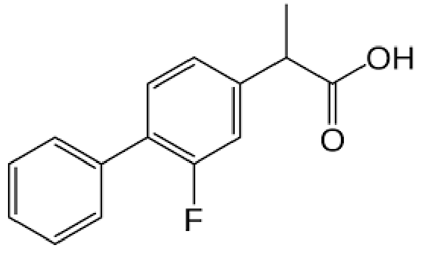	12 [28]	4.16

[*]- pubchem.ncbi.nlm.nih.gov.

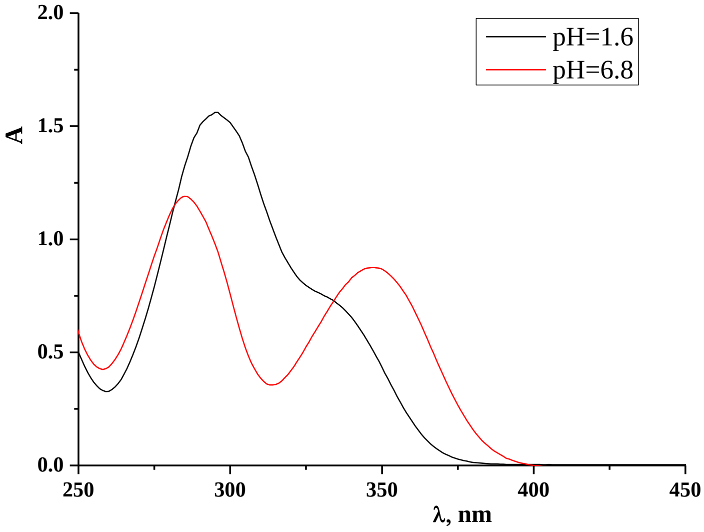

Figure S1. UV/vis spectra of TA in different buffers

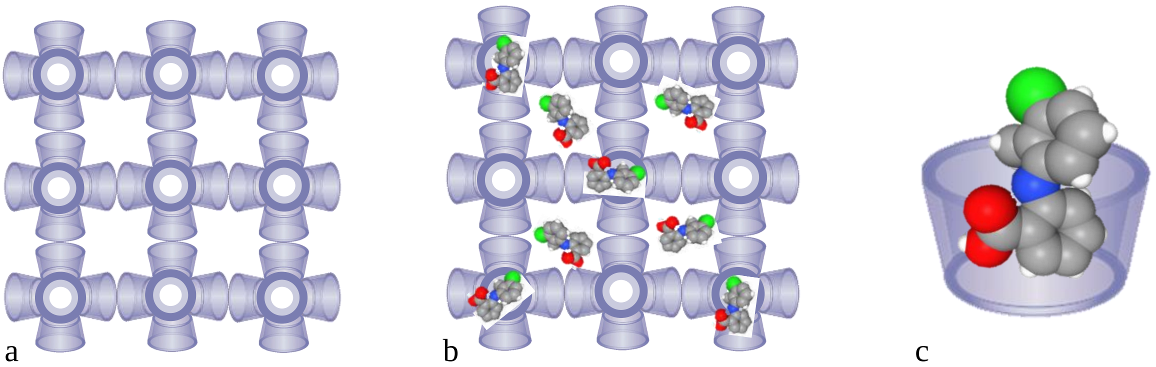

Figure S2. Schematic representation of γCD-MOF (a), γCD-MOF/TA (b) and inclusion complex γ-CD/TA (c).

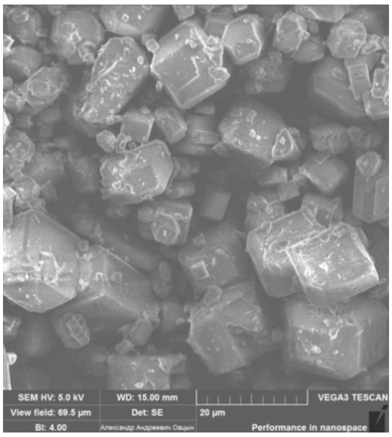

a

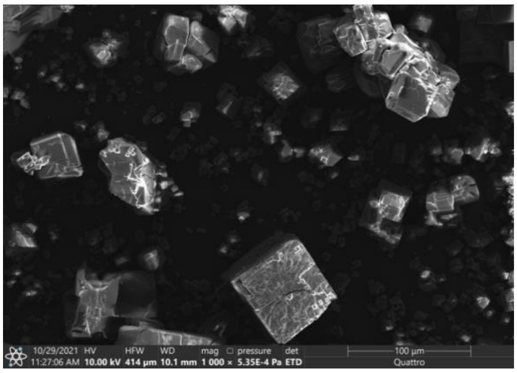

b

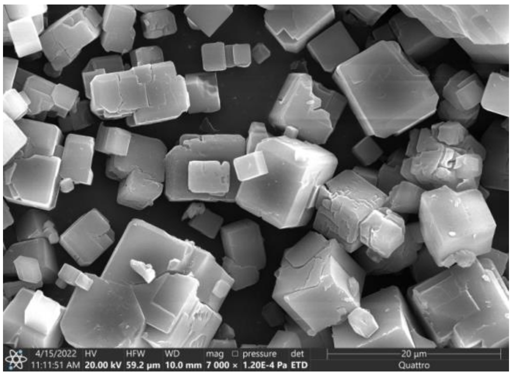

c

Figure S3. SEM images of γCD-MOF (a), γCD-MOF/TA-1 (b) and γCD-MOF/TA-2 (c).

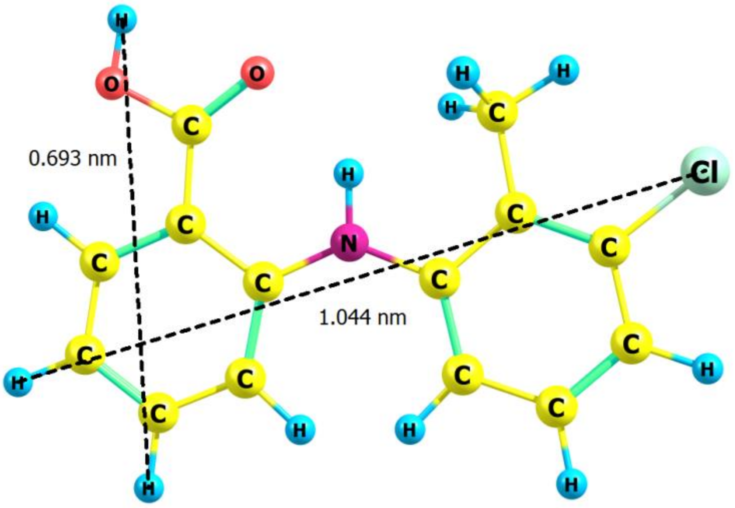

Figure S4. Geometric dimensions of TA.
